# Supplementary material for: Identification of photoperiod-induced specific miRNAs in the adrenal glands of Sunite sheep (Ovis aries)
Source: Front Vet Sci. 2022 Jul 22;9:888207. doi: 10.3389/fvets.2022.888207 (PMC9354845; doi:10.3389/fvets.2022.888207)
Supplement: Supplementary file 1 [file Data_Sheet_1.ZIP › Supplementary file/miRDeep2 file.zip!/miRDeep2 file/Novel_Structure/SP42A2/Novel_658.pdf]

Provisional ID	:	NC_019484.2_43500
Score total	:	1.9
Score for star read(s)	:	-1.3
Score for read counts	:	0
Score for mfe	:	1.6
Score for randfold	:	1.6
Score for cons. seed	:	
Total read count	:	536
Mature read count	:	536
Loop read count	:	0
Star read count	:	0

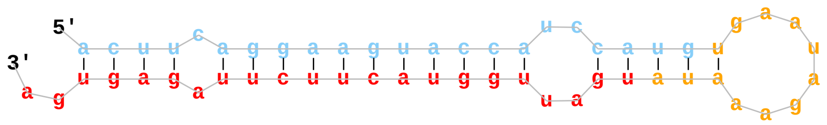

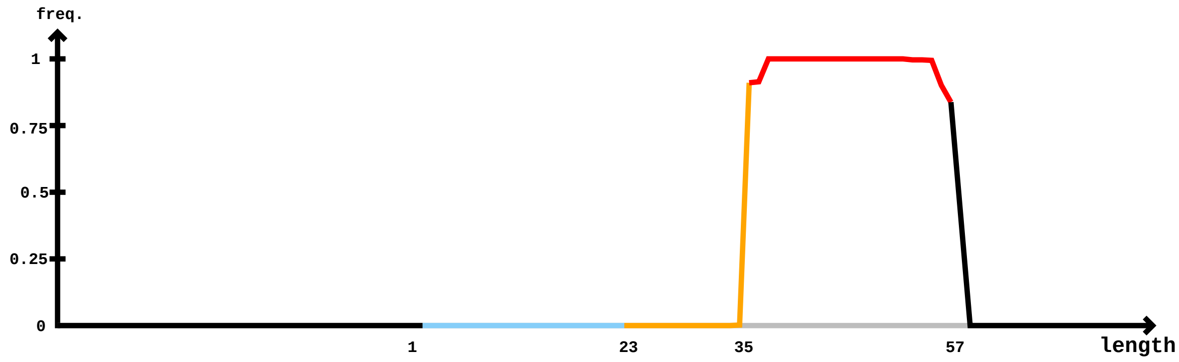

Star Mature

```
5'- ggucaccacauuucacuaugccgcgugcaguauugcacuucaggaaguaccauccaugugaauagaaauaugauugguacuucuuagagugaaguaaugcauagcaaauaca   -3'    exp
    (((...............)))(((((((.((((.(((((.((((((((((..(((((........)))))..)))))))))).))))).)))).))))).))..........    reads   mm   sample
    .....................................................................augauugguacuucuuagagu.....................    1       0    seq
    ......................................................................ugUuugguacuucuuag.........................    1       1    seq
    ......................................................................ugauugguacuucuuag.........................    1       0    seq
    ......................................................................ugauugguacuucuuagag.......................    1       0    seq
    ......................................................................ugauugguacuucuuagagu......................    49      0    seq
    ......................................................................ugauugguacuucuuagagug.....................    34      0    seq
    ......................................................................ugauugguacuucuuagaguga....................    376     0    seq
    ......................................................................ugauugguacuucuuagagugaa...................    25      0    seq
    .......................................................................gauugguacuucuuagaguga....................    2       0    seq
    ........................................................................auugguacuucuuagaguga....................    31      0    seq
    ........................................................................auugguacuucuuagagugaa...................    15      0    seq
```
